# Supplementary material for: Sample-efficient inverse design of freeform nanophotonic devices with physics-informed reinforcement learning
Source: Nanophotonics. 2024 Feb 27;13(8):1483–92. doi: 10.1515/nanoph-2023-0852 (PMC11636486; doi:10.1515/nanoph-2023-0852)
Supplement: Supplementary file 1 — Supplementary Material Details [file j_nanoph-2023-0852_suppl_001.pdf]

Supplementary Materials for

Sample-efficient inverse design of freeform nanophotonic devices with physics-informed reinforcement learning

Chaejin Park[1,2†], Sanmun Kim[1†], Anthony W. Jung[2,†], Juho Park[1], Dongjin Seo[1,3], Yongha Kim[2], Chanhyung Park[1], Chan Y. Park[2*], Min Seok Jang[1*]

[1]School of Electrical Engineering, Korea Advanced Institute of Science and Technology, Daejeon 34141, Republic of Korea

[2]KC Machine Learning Lab, Seoul 06181, Republic of Korea

[3]AI Team, Glorang Inc., Seoul 06140, Republic of Korea

Correspondence to:
* chan.y.park@kc-ml2.com
* jang.minseok@kaist.ac.kr

†These authors contributed equally to this work.

This PDF file includes:

Supplementary Text
Figs. S1 to S6
Tables. S1 to S8

S1. Network architecture

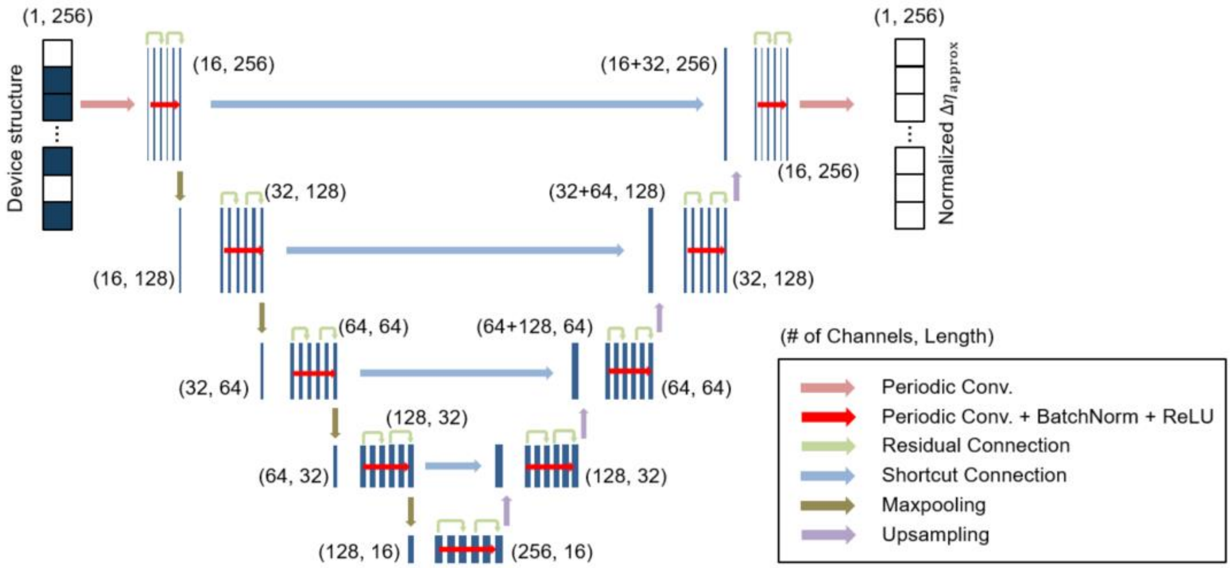

Figure S1. Neural network architecture of the agent. Number of channels and vector sizes are indicated as tuples.

S2. Training dataset configuration

In the pre-training stage of PIRL, we use a dataset consisting of 20,000 structure-adjoint gradient pairs. The number of training dataset is selected based on the tradeoff between the prediction accuracy and the sample efficiency. We run five trials of supervised learning with each training data set consisting of 2,500, 5,000, 10,000, 20,000 and 40,000 samples. The calculated root-mean-square error (RMSE) is normalized with the standard deviation of the adjoint gradients in the training set. Figure S2a shows how the prediction error of the neural network changes as a function of the number of training samples available to the network.

The periodic nature of the system enables data augmentation through transversely displaced samples. The data-augmented training dataset has a size of 640,000. Also, as the samples with exceptionally high adjoint gradients obstruct the supervised learning process, they were excluded from the training dataset.

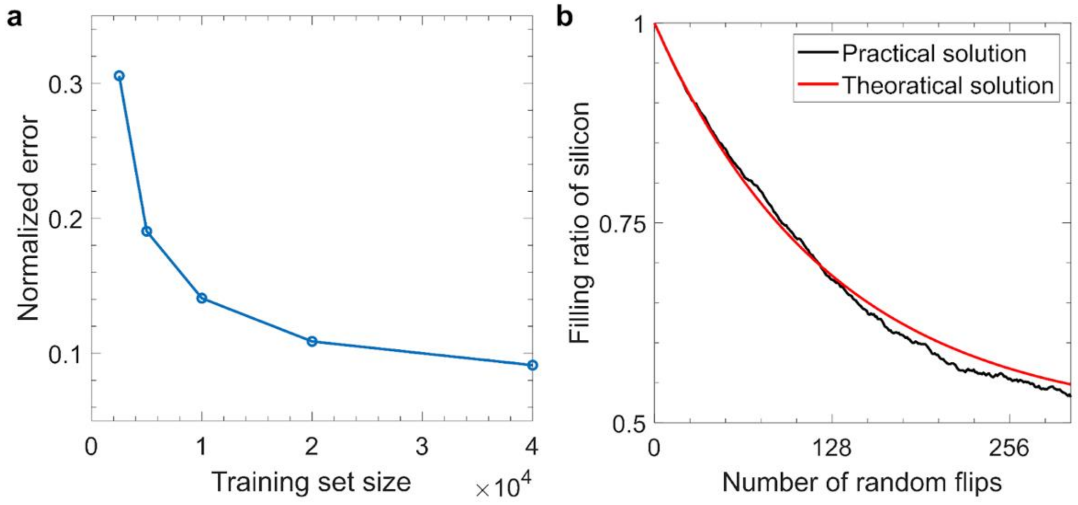

Figure S2. a, The prediction error of the neural network for different training set sizes under a target condition λ = 1100 nm, θ = 60°. Normalized error is calculated by dividing the test set root-mean-square-error (RMSE) by the standard deviation of the training set, ~0.0625. **b, Filling ratio of the Si cells during the random flipping.** Practical solution is estimated based on the Monte Carlo simulation and the theoretical solution is obtained through solving the differential equation $y' = [-y + (1 - y)]/256$, with initial condition $y(0) = 1$, where y represents a filling ratio of silicon cells during the random flipping of cells.

Randomly flipped samples are used to create the dataset for the pretraining stage. We plot the ratio of cells filled with silicon among the 256 cells assuming a full exploration (a fully-random flip). Each episode starts off with a silicon-filled device represented as [1, 1, …, 1]. Figure S2b shows

the filling ratio of silicon as a function of the number of random flips. As the number of random flips increases, the filling ratio of the silicon converges to 50%. The silicon filling ratio of samples in the training dataset was set to follow the distribution given in Figure S2b as RL initially starts off from exploration dominated processes through Epsilon scheduling.

S3. Comparison between the flip-value and the adjoint gradient

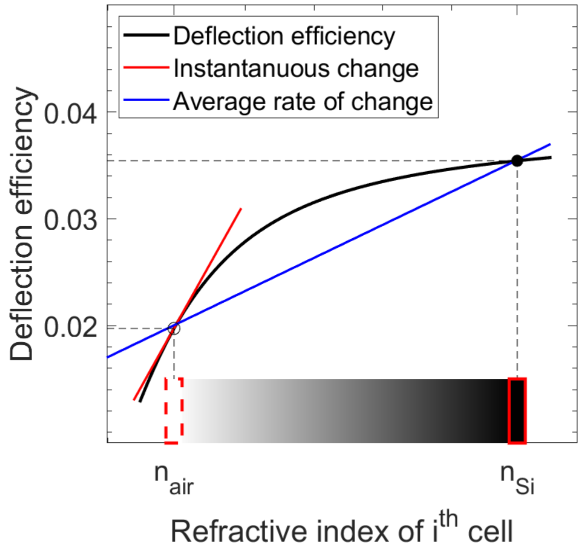

Figure S3. The conceptual explanation of the adjoint gradient and the flip-difference calculation. The deflection efficiency is plotted as a black line in the figure. The adjoint gradient is a local approximation (red) of the average rate of change in the flip-difference calculation (blue).

S4. Algorithm summary

Table S1. Pre-training stage

Algorithm 1 Pre-training stage of PIRL

Require: $\Delta\eta_{\text{approx}}$ calculator $g : s \rightarrow (\frac{\delta\eta}{\delta n}) \cdot \Delta n$, $rand_{flip}$:= random flipping of a cell.

Input: Initial structure $s_{\text{Silicon}} = \{-1, +1, ..., +1\}$ and $s_{\text{Air}} = \{+1, -1, ..., -1\}$, training set size N_{sample}, maximum number of random flipping N_{flip}, approximator f, network parameter θ.

Output: Training samples with size N_{sample}, optimized network parameters θ.

Initialization Current device structure s_0, network parameters ω.
for $1, 2, ..., (N_{sample}/2 \cdot N_{flip})$ **do**
  for $j = 1, 2, ..., N_{flip}$ **do**
    Set initial structure $s_0 = s_{\text{Silicon}}$
    for number of random walk $k = 0, 1, ..., j-1$ **do**
      Take action $a_k = rand_{flip}$ on structure s_k and obtain s_{k+1}
    end for
    Calculate $\Delta\tilde{\eta}_{\text{approx}} = g(s_j)/\|g(s_j)\|_2$
    Store $(s_j, \Delta\tilde{\eta}_{\text{approx}})$ in training set
    Set initial structure $s_0 = s_{\text{Air}}$
    for number of random walk $k = 0, 1, ..., j-1$ **do**
      Take action $a_k : rand_{flip}$ on structure s_k and obtain s_{k+1}
    end for
    Calculate $\Delta\tilde{\eta}_{\text{approx}} = g(s_j)/\|g(s_j)\|_2$
    Store $(s_j, \Delta\tilde{\eta}_{\text{approx}})$ in training set
  end for
end for
Pre-process samples in the training set
if *training stage* **then**
  Randomly sample $(s, \Delta\tilde{\eta}_{\text{approx}})$ from training set
  Calculate loss $L = \text{MSE}(\Delta\tilde{\eta}_{\text{approx}}, f_\theta(s))$
  Update network parameters $\theta \leftarrow \text{Adam}(\theta)$
end if

Table S2. RL stage

Algorithm 2 Agent-environment interaction in a single worker

Require: Deflection efficiency calculator $f : s \rightarrow \eta$, $rand_{flip}$:= random flipping of a cell.

Input: Initial structure $s_0 = \{-1, +1, ..., +1\}$, number of episodes k, network parameters ω, target network parameters ω^-, learning rate α, discount factor γ.

Output: Trajectory $(s, a, r, s^{'})$, optimized network parameters ω.

Initialization Current device structure s_0, target network parameters $\omega^{'} \leftarrow \omega$.
for number of episodes $i = 0, 1, ..., k-1$ **do**
  Set current structure s_i
  Take action a_i : $\begin{cases} \underset{a}{\text{argmax}}\, Q^{\omega}(s_i, a), & \text{with probability } 1\text{-}\epsilon \\ rand_{flip}(s_i), & \text{with probability } \epsilon \end{cases}$
    on structure s_i and obtain s_{i+1}
  Calculate reward $r_i = \Delta\eta = \eta_{i+1} - \eta_i$, where $\eta_{i+1} = f(s_{i+1})$
  Store transition (s_i, a_i, r_i, s_{i+1}) in experience replay
  if *training stage* **then**
    Randomly sample transitions (s_t, a_t, r_t, s_{t+1}) from experience replay
    Calculate loss $L = \text{Huber}(y_t, Q^{\omega}(s, a))$,
    where Bellman target $y_t = r_t + \gamma \max_{a} Q^{\omega^-}(s_{t+1}, a_{t+1})$
    Update network parameters $\omega \leftarrow \text{Adam}(\omega)$
  end if
  if *update target network* **then**
    $\omega^- \leftarrow \omega$
  end if
end for

Table S3. Genetic algorithm

**Variable name**	**Value**
MaxGeneration	400
PopulationSize	500

We used the genetic algorithm module provided in the Global Optimization Toolbox in MATLAB. Table S3 outlines the value of the hyperparameters used. Other hyperparameters are unchanged from their default values. Outline of each variable can be found in the following link: [1]

Table S4. Greedy algorithm

Algorithm 3 Greedy depth 1 algorithm

Require: Deflection efficiency calculator $f : s \to \eta$.

Input: Randomly selected initial structure $s_0 \in [-1, 1]^N$ where N is number of cells, action of flipping a cell in i^{th} index $a(i)$, maximum number of iterations k. *tolerance* determines early stop condition.

Output: Best device structure s_{best}, and its efficiency $\eta_{\max} = f(s_{\text{best}})$ after $256 \times k$ simulations.

Initialize $s_{\text{best}} = s_0$, $\eta_{\max} = 0$, $stop = 0$
while $i \leq k$ **do**
  Set current structure $s_i = s_{\text{best}}$
  for cell index $j = 0, 1, ..., N-1$ **do** {search for the best of all possible actions}
    Take $a(j)$ on structure s_i and obtain $s_i^{(j)}$
    Calculate deflection efficiency $\eta^{(j)} = f(s_i^{(j)})$
    Collect structure $s_i^{(j)}$ and efficiency $\eta^{(j)}$
  end for
  Take action $a_{\text{opt}}(k)$ where $k = \underset{j}{\text{argmax}}\, \eta^{(j)}$ and obtain $s_i^{a_{\text{opt}}}$, $\eta^{a_{\text{opt}}}$
  if $\eta_{\max} < \eta^{a_{\text{opt}}}$ **then**
    Update best structure $s_{\text{best}} = s_i^{a_{\text{opt}}}$ and max efficiency $\eta_{\max} = \eta^{a_{\text{opt}}}$
    $stop = 0$
  end if
  $i = i + 1$
  $stop = stop + 1$
  if $stop \geq tolerance$ **then**
    break
  end if
end while

Table S5. Random search

Algorithm 4 Random search algorithm

Require: Deflection efficiency calculator $f : s \to \eta$, $rand :=$ a random device generator.

Input: Randomly selected initial structure $s_0 \in [-1, 1]^N$ where N is number of cells, maximum number of iterations k.

Output: Best device structure s_{best}, and its efficiency $\eta_{\max} = f(s_{\text{best}})$ after k simulations.

Initialize $s_{\text{best}} = s_0$, $\eta_{\max} = 0$
while $i \leq k$ **do**
  Obtain random structure $s_i = rand$
  Calculate deflection efficiency $\eta = f(s_i)$
  if $\eta_{\max} < \eta$ **then**
    Update best structure $s_{\text{best}} = s_i$ and max efficiency $\eta_{\max} = \eta$
  end if
  $i = i + 1$
end while

We set the *tolerance* parameter = 5.

S5. Hyperparameter table of reinforcement learning

All hyperparameters used in the RL stage are provided in Table S6. Description of each hyperparameter can be found on the RLlib website [2].

Table S6. List of hyperparameters in RL

**Variable name**	**Value**
overall learning steps	2×10^5
learning_starts	1,000
target_network_update_freq	2,000
buffer capacity	10^5
learning rate	0.001
gamma	0.99
training batch size	512
number of training	1
horizon	512
number of rollout workers	16
number of environments per workers	1
initial epsilon	0.99
final epsilon	0.01
epsilon timesteps	100,000

S6. How RL avoids the local minima problem

Many optimization methods including the adjoint gradient method suffer from the problem of falling into local minima. PIRL mitigates this issue by training a deep network during the RL stage. In theory, applying the Bellman operator over an infinite number of times leads to an optimal Q function for any arbitrary initialization [3]. Since evaluating optimal Q value requires sweeping over the entire state space, which is infeasible, a neural network is adopted to approximate the optimal Q function. This approximation is refined through stochastic gradient descent (SGD). Previous theoretical investigations indicate that when a neural network is sufficiently parameterized, SGD converges to the global minimum [3]. Additionally, in our algorithm, a high rate of ε-greedy exploration is set at the beginning of training, to refrain the agent from being trapped in a local optima.

S7. Ablation study for the physics-informed pre-training and the neural network architecture of the RL agent

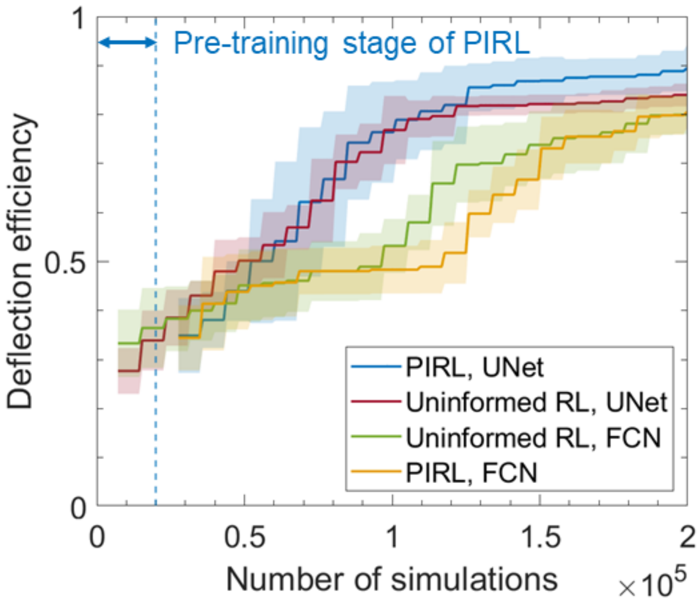

Figure S4. Optimization curve showing the maximum values of deflection efficiency, obtained using U-Net based PIRL (blue), U-Net based uninformed RL (red), fully connected network (FCN) based uninformed RL (green), FCN based PIRL (yellow) under the target condition λ = 1100 nm, θ = 60°.

Table S7. The maximum, average, and standard deviation of final devices from RL-based algorithms under the target condition λ = 1100 nm, θ = 60°.

	Max	Mean ± Stdev
PIRL, UNet	94.9	89.4 ± 4.6
PIRL, FCN	87.0	80.7 ± 3.2
Uninformed, Unet	87.1	84.0 ± 2.3
Uninformed, FCN	88.8	82.8 ± 3.1

S8. Average time consumption of each optimization algorithm

Table S8. Average time consumption of each optimization algorithm.

Step		Total # of devices	Total cost (hours)
PIRL, UNet	Pre-training stage	20,000	0.8 h
	RL stage	180,224	1.2 h
PIRL, FCN	Pre-training stage	20,000	0.05 h
	RL stage	180,224	0.8 h
Uninformed RL, UNet	RL stage	200,704	1.2 h
Uninformed RL, FCN	RL stage	200,704	0.7 h

S9. E-field distribution of an optimized device at a different target condition

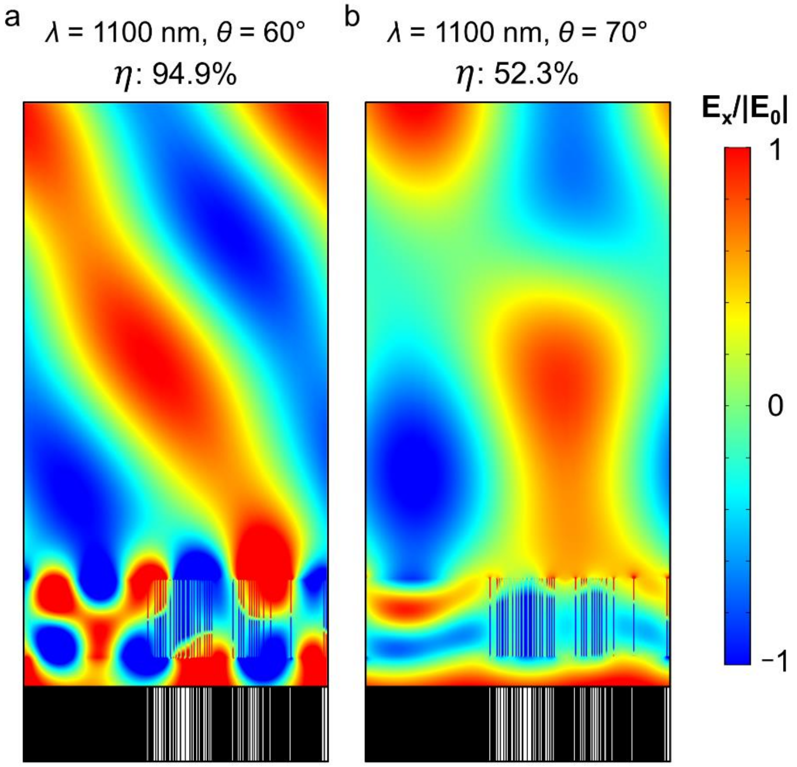

Figure S5. Electric field distribution from an optimized beam deflector at (1100 nm, 60°) operating at a different target condition (1100 nm, 70°). a, Device with the same state representation in Figure 4a. **b,** The structure shows deflection efficiency of 52.3% at (1100 nm, 70°) whereas the deflection efficiency at the original condition is 94.9%.

S10. Transfer learning results: Mean and standard deviation

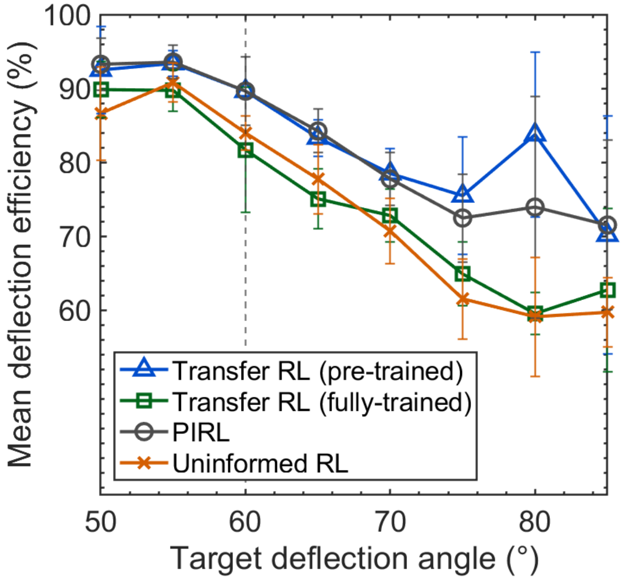

Figure S6. The average deflection efficiency of the device found using PIRL (gray), uninformed RL (orange), and two different transfer learning processes: pre-trained model transfer RL (blue), and fully-trained model transfer RL (green). The data points and error bars represent the averages and standard deviations of maximum efficiencies from ten runs, respectively.

References

1. *https://nl.mathworks.com/help/gads/options-in-genetic-algorithm.html*.
2. *https://docs.ray.io*.
3. J. Fan et al. "A theoretical analysis of deep Q-learning." Learning for dynamics and control. PMLR (2020).
